# Outcomes of Children With Firearm Injuries Admitted to the PICU in the United States*

**DOI:** 10.1097/PCC.0000000000002785

**Published:** 2021-06-07

**Authors:** Dayanand Bagdure, Cortney B. Foster, Nan Garber, Adrian Holloway, Jenni Day, Jessica Lee, Gerardo Soto-Campos, Nancy Brundage, Adnan Bhutta, Ana Lia Graciano

**Affiliations:** 1 Department of Pediatrics, Division of Critical Care Medicine, University of Maryland School of Medicine, Baltimore, MD.; 2 University of Maryland Medical Center, Baltimore MD.; 3 Graduate Medical Education, University of Maryland School of Medicine, Baltimore MD.; 4 Virtual Pediatric Systems, LLC, Los Angeles, CA.

**Keywords:** critical care, firearm injuries, outcomes, pediatric intensive care unit, pediatrics, United States

## Abstract

Firearm-related injury is the second leading cause of injury and death for children 1–18 years old in United States. The objective of our study was to analyze the outcomes of children admitted to the PICU with firearm injuries.

**DESIGN:**

Retrospective study.

**SETTING:**

PICUs in United States contributing data to Virtual Pediatric Systems, LLC, from January 2009 to December 2017.

**PATIENTS:**

Children age 1 month to 18 years old admitted to the PICU with firearm injury, identified by external cause of injury E-codes and *International Classification of Diseases*, 9th Edition, and *International Classification of Diseases*, 10th Edition, codes were identified.

**INTERVENTIONS:**

None.

**MEASUREMENTS AND MAIN RESULTS:**

There were 1,447 cases identified of which 175 (12%) died in the PICU. Unintentional firearm injury (67.7%) and assault with a firearm injury (20%) comprised 90% of the cases. Males comprised 78% of the cohort (1,122) and race distribution included 45% Black (646), 27% White (390), and 12% Hispanic (178). Among the children who died in the PICU, 55% were 13–18 years old. Children attempting suicide with a firearm were more likely to die in the PICU as compared to the other causes of firearm injury. Based on their Pediatric Overall Performance Category and Pediatric Cerebral Performance Category scores at discharge, there is high morbidity in children with firearm injuries.

**CONCLUSIONS:**

Mortality rate of children with firearm injury admitted to the PICU is high. Children admitted to the PICU with suicide attempt with a firearm carried the highest mortality. Further studies may help further define the epidemiology of firearm injuries in children and plan interventions to minimize these unnecessary deaths.

## Abstract 2

Supplemental Digital Content is available in the text.

Firearm injuries have risen to become the second leading cause of death in the United States for children and adolescents 1–18 years old (1, 2). Nationally, it is estimated that nearly 20,000 children and adolescents are seen in emergency rooms annually due to firearm-related injuries, and more than 7,000 are hospitalized (3–5). Using Pediatric Health Information System database, Kamat et al (6) observed an increase in median cost per hospitalization and increase in critical care resources due to firearm injuries, with mortality of 13.2%. The purpose of this study is to understand the burden and mortality outcomes of children admitted with firearm injuries using a larger PICU database.

## MATERIAL AND METHODS

Data were obtained from Virtual Pediatric Systems (VPS) LLC, with 135 PICUs contributing and participating in data gathering and analysis (7). A total of 107 sites provided data from which 101 were trauma sites (level 1: 71, level 2: 27, level 3: 3, and not applicable: 6). The external cause of injury codes (E-codes) from the *International Classification of Diseases* (ICD), 9th Edition, and ICD, 10th Edition, listings were used to identify the cases, and only the primary admission data were obtained. Once identified, the cases were grouped according to the intent of injury: assault, suicide, unintentional, and undetermined cause. The rationale to classify them in such a manner was to analyze if the outcomes in the PICU vary based on the intent of injury.

We obtained patient demographic data on gender, race, and age. Mortality was calculated when the death occurred in the PICU. Mortality was the primary outcome measured, and Pediatric Overall Performance Category (POPC) and Pediatric Cerebral Performance Category (PCPC) scores were the secondary outcomes. These validated scores are used to describe the short-term outcomes of children by quantifying the overall functional morbidity and cognitive impairment (8–10).

The study was approved by the Institutional Review Board of the University of Maryland, Baltimore. The study data included children 1 month to 18 years old admitted with firearm injuries between January 1, 2009, and December 31, 2017.

All data were entered into IBM SPSS Statistics for Windows, Version 24.0 (Armonk, NY: IBM Corp. Released 2016).

## RESULTS

**Table 1** describes the demographic data for our cohort, and of the 1,447 cases, 1,272 (88%) survived, and 175 (12%) died. Higher mortality was observed in White children compared with Black children and Hispanic children (41.1%, 30.9%, 6.9%, respectively; *p* < 0.05). Mortality was highest in the suicide category as compared to the other types of injuries with firearms (*p* < 0.05), with head/neck injury as the most common site (30.2%). One third of the children (33.5%) with head/neck category died in the PICU.

**TABLE 1.** Demographic Data for Children With Firearm Injuries Admitted to the PICU from VPS Database 2009–2017

Variables	Survived (N = 1,272)	Died (N = 175)	Total (N = 1,447)	χ2 Tests of Independence
Gender, *n* (%)				χ^2^ (1) = 0.064
Female	287 (22.6)	38 (21.7)	325 (22.4)	*p* = 0.801
Male	985 (77.4)	137 (78.3)	1,122(77.5)	*n* = 1,447
Race, *n* (%)				χ^2^ (5) = 57.3
Black or African American	592 (46.5)	**54 (30.9**)^a^	646 (44.6)	*p* < 0.05
White	318 (25.0)	**72 (41.1**)^c^	390 (26.9)	*n* = 1,324
Hispanic	**166 (13.1**)^a^	12 (6.9)	178 (12.3)	
Asian/Indian/Pacific Islander	17 (1.3)	0	17 (1.17)	
American Indian or Alaska Native	10 (0.8)	0	10 (0.6)	
Hawaiian or other Pacific Islander	1 (0.1)	0	1 (0.06)	
Other/mixed	43 (3.4)	7 (4.0)	50 (3.4)	
Unspecified	19 (1.5)	**13 (7.4**)^c^	32 (2.2)	
Missing data	106 (8.3)	17 (9.7)	123 (8.5)	
Age, *n* (%)				χ^2^ (3) = 4.99
1–23 mo	42 (3.3)	9 (5.1)	51 (3.5)	*p* = 0.173
2–5 yr	178 (14)	17 (9.7)	195 (13.4)	*n* = 144
6–12 yr	319 (25.1)	52 (29.7)	371 (25.6)	
13–18 yr	733 (57.6)	97 (55.4)	830 (57.3)	
Cause of injury, *n* (%)				χ^2^ (3) =171.22
Unintentional	887 (69.7)	**93 (53.1**)^a^	980 (67.7)	*p* < 0.05
Suicide attempt	**43 (3.4**)^b^	**52 (29.7**)^c^	95 (6.5)	*n* = 1,420
Assault	267 (21.0)	**24 (13.7**)^a^	291 (20.1)	
Undetermined	49 (3.9)	5 (2.9)	54 (3.7)	
Missing data	25 (2.0)	1 (0.6)	26 (1.7)	
Site of injury, *n* (%)				χ^2^ (5) = 94.3
Head/neck injury	328 (25.7)	**110 (62.8**)	438 (30.2)	*p* < 0.05
Chest injury	195 (15.3)	8 (4.5)	203 (14.0)	*n* = 1,292
Abdominal injury	183 (14.3)	1 (0.5)	184 (12.7)	
Spine injury	58 (4.5)	1 (0.5)	59 (4.0)	
Extremity injury	96 (7.5)	0	96 (6.6)	
Missing	119 (9.3)	**16 (9.1**)	135 (9.3)	
Undetermined	293 (23.0)	39 (22.2)	332 (22.9)	

^a^Standardized residual –2.0 to –2.9.

^b^Standardized residual ≥ –3.0.

^c^Standardized residual ≥ 3.0.

Boldface values indicate statistical significance.

**Table 2** shows the most common cause of injury was unintentional (67.7%). Assault by firearm was disproportionally higher in Black children (65%), whereas suicide attempt was predominantly in White children (63%) (*p* < 0.05). Table 2 shows the POPC and PCPC scores. Increases in mild, moderate, or severe overall disability show an impact on overall functional outcome. When using the POPC score, most discharged children had mild disability. Children injured due to undetermined cause had the largest decline in POPC scores. Only 10.3% of the total patients had good overall performance at the time of discharge (**Supplemental Table**, http://links.lww.com/PCC/B807).

**TABLE 2.** Data for Children With Firearm Injuries According to the Type of the Injury for the Years 2009–2017

Variables (*N* = 1,420)	Unintentional (*N* = 980)		Suicide Attempt (*N* = 95)		Assault (*N* = 291)		Undetermined (*N* = 54)		χ^2^ Tests of Independence
Outcome, *n* (%)									*p* < 0.05
Survived	887 (90.5)		43 (45.3)^c^		267 (91.8)		49 (90.7)		
Died	93 (9.5)^b^		52 (54.7)^e^		24 (8.2)^b^		5 (9.3)		
Gender, *n* (%)									*p* = 0.337
Female	229 (23.4)		15 (15.8)		65 (22.3)		10 (18.5)		
Male	751 (76.6)		80 (84.2)		226 (77.7)		44 (81.5)		
Race, *n* (%)									*p* < 0.05
Black or African American	413 (42.1)		6 (6.3)^c^		189 (64.9)^e^		24 (44.4)		
White	285 (29.1)		60 (63.2)^e^		25 (8.6)^c^		11 (20.4)		
Hispanic	112 (11.4)		10 (10.5)		44 (15.1)		11 (20.4)		
Asian/Indian/ Pacific Islander^a^	9 (0.9)		1 (1.1)		6 (2.1)		1 (1.9)		
American Indian or Alaska Native^a^	8 (0.8)		0		2 (0.7)		0		
Other/mixed^a^	32 (3.3)		4 (4.2)		12 (4.1)		1 (1.9)		
Unspecified	20 (2.0)		6 (6.3)^d^		2 (0.7)		4 (7.4)^d^		
Missing data	101 (10.3)		8 (8.4)		11 (3.8)		2 (3.7)		
Age, *n* (%)									*p* < 0.05
1–23 mo	40 (4.1)		0		11 (3.8)		0		
2–5 yr	170 (17.3)^e^		0^d^		17 (5.8)^c^		4 (7.4)		
6–12 yr	295 (30.1)^d^		7 (7.4)^c^		48 (16.5)^c^		14 (25.9)		
13–18 yr	475 (48.5)^c^		88 (92.6)^e^		215 (73.9)^e^		36 (66.7)		
Arterial catheter placement, *n* (%)	485 (49.5)		70 (73.7)^e^		149 (51.2)		29 (53.7)		*p* < 0.05
CT scan, *n* (%)	154 (15.7)		12 (12.6)		53 (18.2)		7 (13.0)		*p* < 0.05
Craniotomy/craniectomy, *n* (%)	92 (9.4)		11 (11.6)		16 (5.5)		3 (5.6)		*p* < 0.05
Intracranial pressure monitoring/extraventricular drain/Licox, *n* (%)	87 (8.9)		16 (16.8)^d^		21 (7.2)		4 (7.4)		*p* < 0.05
Pediatric Overall Performance Category scores (%)	Baseline	Discharge	Baseline	Discharge	Baseline	Discharge	Baseline	Discharge	
Death	0	22.9	0	61.4	0	12.7	0	8.3	
Coma or vegetative state	1.7	1.9	0	0	0.9	0.8	0	0	
Severe overall disability	1.3	6	0	2.3	0	5.1	0	5.6	
Moderate overall disability	6.1	15	1.1	13.6	1.7	22	5.9	16.7	
Mild overall disability	4.4	23.3	12.1	11.4	6	24.6	17.6	61.1	
Good overall performance	86.5	30.8	84.8	11.4	91.4	34.7	76.5	83	
Pediatric Cerebral Performance Category scores (%)	Baseline	Discharge	Baseline	Discharge	Baseline	Discharge	Baseline	Discharge	
Death	0	22.8	0	61.4	0	12.7	0	8.3	
Coma or vegetative state	1.7	1.9	0	0	0.9	0.8	0	0	
Severe overall disability	0.4	1.9	0	2.3	0	2.5	0	0	
Moderate overall disability	3.1	5.6	0	6.8	0	3.4	0	2.8	
Mild overall disability	2.6	6.4	3	4.5	3.4	8.5	8.8	11.1	
Normal	92.1	61.4	97	25	95.7	72	91.2	77.8	
Invasive mechanical ventilation, *n* (%)	496 (50.6)		74 (77.9)^e^		148 (50.9)		19 (35.2)		*p* < 0.05
Central venous catheter, *n* (%)	289 (29.5)		57 (60.0)^e^		79 (27.1)		16 (29.6)		*p* < 0.05
Total duration of intubation, mean (sd)	1.7 (3.72)		1.9 (3.36)		1.73 (4.22)		1.36 (3.29)		p < 0.05

^b^Standardized residual –2.0 to –2.9.

^c^Standardized residual ≥ –3.0.

^d^Standardized residual 2.0–2.9.

^e^Standardized residual ≥ 3.0.

Twenty-six missing cause of injury. Bold numbers indicate statistical significance. “Common procedure”(> 50 times) represent only 3.4% of the cohort and increasing to 10% threshold; craniotomy and ICP monitor would not be included, but were included for the clinical significance.

## DISCUSSION

Important findings of our study include that almost two thirds of the cases in the PICU were due to unintentional firearm injury, admission to the PICU with firearm injury carried high mortality, and head/neck injury with a firearm was a risk factor for death.

Overall, the mortality rate of children admitted to the PICU for all causes is low. In comparison, the mortality rate due to firearm injuries in our study was 12% (*n* = 175), more than five times the all cause ICU mortality (11). There are very few PICU disease processes that carry such a high mortality rate. Interstage single ventricle physiology (12%, 30 d mortality) (12, 13), oncology patients with sepsis (17%) (14), or severe pediatric acute respiratory distress syndrome (33%) (15) are few notable exceptions.

Unintentional injury was common and carried high mortality, with 50% of the children being between 13 and 18 years old. As most of the patients with firearm injury are due unintentional injuries, prevention strategies can significantly decrease this burden in the PICU. Reducing the burden of unintentional injuries should provide a target for societal (policy and advocacy) interventions. It is known that males disproportionately bear the burden of firearm injury and that there are significant differences between older and younger children in terms of intent (intentional vs unintentional) and mortality, with the highest mortality in those greater than 13 years old who attempted suicide (1).

Children attempting suicide with a firearm were more likely to die in the PICU, compared with other categories of firearm injury. Although Black children had the highest rates of firearm injury, White children had the highest rate of suicide and as a subgroup had a significantly higher mortality. We also find that head/neck injury, common in suicide attempts, had the highest mortality. These studies need wider dissemination to design effective preventive strategies to combat the high mortality in children attempting suicide (16).

In addition to the high mortality, survivors had a wide range of morbidities and neurologic disabilities as demonstrated by the POPC and PCPC (Table 2). A significant decline was found upon discharge, and only 10.3% of patients had good overall performance. The POPC and PCPC scores compared with baseline at discharge reveal significant morbidity among victims of firearm injuries indicating that even after survival, a significant number of patients are likely to require rehabilitation services. The disposition of the survivors (1,272) was as follows: 982 (77%) to the floor, 81 (6.3%) home, 145 (11.3%) to a step down unit/telemetry/technology dependent unit, and 33 (2.5%) to a rehabilitation facility.

Our study’s limitations include errors in coding, which is inherent to any study based on databases. VPS maintains the accuracy of data by quality check measures and appropriate training of the staff. The data from VPS cannot be generalized as it has not been standardized to national estimates. Pediatric deaths due to firearms outside of the hospital or in the emergency rooms are not included in this database.

Gani et al (17) studied the nationwide emergency department (ED) sample finding about 35% of all ED visits with firearm injury result in inpatient admission. The overall mortality for their cohort was 6%, and interestingly, the ED mortality was lower (3.6%) than the inpatient mortality (6.6%). Similar to a recent study (6), the higher mortality in our cohort could be because of one of the following reasons. Academic centers, which are more likely to contribute data to the VPS database, are the tertiary centers for pediatric patients and tend to have more acuity and complexity.

Children with firearm injuries admitted to the PICU represent a very small cohort of the whole PICU population. Most of the firearm injuries are nonfatal and do not require PICU admission. Nevertheless, morbidity and mortality in the PICU give a measure of the impact on families and healthcare.

## CONCLUSIONS

Our study using data from VPS reports that firearm injury is associated with increased morbidity including neurologic injury evidenced by POPC and PCPC scores and with five times the mortality as the comparable general PICU population. With advances in health informatics and increasing collaboration to form national registries and databases, it has become feasible to better understand the impact of health conditions, like firearm injuries on the society. Accurate epidemiologic data can aid in creating policies that can be used to advocate for pediatric victims of firearm violence.

## Supplementary Material


