# Supplementary material for: Outcomes of Children With Firearm Injuries Admitted to the PICU in the United States*
Source: Pediatr Crit Care Med. 2021 Jun 7;22(11):944–9. doi: 10.1097/PCC.0000000000002785 (PMC8565509; doi:10.1097/PCC.0000000000002785)
Supplement: Supplementary file 1 [file pcc-22-0944-s001.docx]

| POPC (%) | Death | | Coma or Vegetative | | Severe overall  disability | | Moderate overall disability | | Mild overall disability | | Good overall performance | |
| --- | --- | --- | --- | --- | --- | --- | --- | --- | --- | --- | --- | --- |
|  | B | D | B | D | B | D | B | D | B | D | B | D |
| Head/neck | 0 | 39.8 | 1.7 | 5.3 | 0 | 9.7 | 0 | 14.6 | 13.8 | 22.8 | 84.5 | 7.8 |
| Chest | 0 | 3.9 | 0 | 5.2 | 0 | 7.2 | 0.9 | 10.5 | 0 | 13.7 | 97.1 | 59.1 |
| Abdominal | 0 | 0.5 | 0 | 2.7 | 0 | 4.3 | 0 | 8.15 | 4.3 | 15.7 | 95.6 | 68.4 |
| Spine | 0 | 1.6 | 0 | 5.0 | 0 | 10.1 | 0 | 13.5 | 0 | 30.5 | 98.3 | 38.9 |
| Extremity | 0 | 0 | 0 | 0 | 0 | 0 | 0 | 11.1 | 0 | 18.9 | 100 | 80 |
| Missing | 0 | 11.8 | 1.4 | 3.8 | 0 | 8.8 | 0 | 11.2 | 3.7 | 20.7 | 94.0 | 43.7 |
| Undetermined | 0 | 11.7 | 0 | 5.4 | 0 | 7.8 | 0 | 8.4 | 0 | 17.4 | 100 | 49.0 |

Supplemental Table: Percentage of children with firearm injuries with site of injury and POPC categories

-B: POPC at baseline, D- POPC at discharge
